# Antibiotic prescribing in public and private practice: a cross-sectional study in primary care clinics in Malaysia

**DOI:** 10.1186/s12879-016-1530-2

**Published:** 2016-05-17

**Authors:** Norazida Ab Rahman, Cheong Lieng Teng, Sheamini Sivasampu

**Affiliations:** Healthcare Statistics Unit, Clinical Research Centre, 3rd Floor MMA Building, 124 Pahang Road, 53000 Kuala Lumpur, Malaysia; Department of Family Medicine, International Medical University, Seremban, Malaysia

**Keywords:** Anti-bacterial agents, Antibiotic stewardship, Drug prescriptions, Primary healthcare, Private practice, Public health practice

## Abstract

**Background:**

Antibiotic overuse is driving the emergence of antibiotic resistance worldwide. Good data on prescribing behaviours of healthcare providers are needed to support antimicrobial stewardship initiatives. This study examined the differences in antibiotic prescribing rates of public and private primary care clinics in Malaysia.

**Methods:**

We used data from the National Medical Care Survey (NMCS), a nationwide cluster sample of Malaysian public and private primary care clinics in 2014. NMCS contained demographic, diagnoses and prescribing from 129 public clinics and 416 private clinics. We identified all encounters who were prescribed antibiotic and analyse the prescribing rate, types of antibiotics, and diagnoses that resulted in antibiotic.

**Results:**

Five thousand eight hundred ten encounters were prescribed antibiotics; antibiotic prescribing rate was 21.1 % (public clinics 6.8 %, private clinics 30.8 %). Antibiotic prescribing was higher in private clinics where they contributed almost 87 % of antibiotics prescribed in primary care. Upper respiratory tract infection (URTI) was the most frequent diagnosis in patients receiving antibiotic therapy and accounted for 49.2 % of prescriptions. Of the patients diagnosed with URTI, 46.2 % received antibiotic treatment (public 16.8 %, private 57.7 %). Penicillins, cephalosporins and macrolides were the most commonly prescribed antibiotics and accounted for 30.7, 23.6 and 16.0 % of all antibiotics, respectively. More recently available broad-spectrum antibiotics such as azithromycin and quinolones were more frequently prescribed in private clinics.

**Conclusions:**

Antibiotic prescribing rates are high in both public and private primary care settings in Malaysia, especially in the latter. This study provides evidence of excessive and inappropriate antibiotic prescribing for self-limiting conditions. These data highlights the needs for more concerted interventions targeting both prescribers and public. Improvement strategies should focus on reducing inappropriate prescribing.

## Background

Antibiotic resistance is a globally significant threat to public health [1]. A recent report from the World Health Organization (WHO) documented high rates of resistance across a range of bacteria that cause common healthcare-associated and community-acquired infections [2]. Antibiotic resistance is directly related to the rate of antibiotic use in the community [3–5]. There is, however, surprisingly little national data on non-hospital (community) antibiotic use. Furthermore, the data that are available are from studies in high-income countries [4, 5]. Descriptive national data on community antibiotic use from low- and middle-income countries, however, is essential for understanding where over-prescribing of antibiotics may be occurring and the patient and provider characteristics that are associated with higher rates of antibiotic prescription. Understanding these characteristics will help to inform regulatory and health education strategies.

Where data are available on low- and middle-income countries’ antibiotic use in the community, they are usually at a sub-national level. For example, Hadi et al., in their systematic review of non-hospital antibiotic use, identified 11 studies from ten countries [6]. Only one of those studies was related to national data, and that study lacked linked patient characteristics and provider information. The WHO reported a series of pilot studies on community antibiotic use in India and South Africa [7]. However, these studies were based on five surveillance settings and were not nationally representative and did not include community antibiotic use and patient and provider characteristics associated with antibiotic use. In Malaysia, antibiotics are commonly prescribed by primary care providers and most frequently prescribed for people with respiratory tract infections [8–10]. However, data are available only from small-scale studies of selected primary care clinics in certain areas, which may have led to inaccurate estimates of antibiotic use in primary care. In this paper, we use data from the National Medical Care Survey (NMCS) 2014 to describe the frequency and characteristics of antibiotic prescribing in Malaysian primary care by detailing the type of antibiotics and their indications. These data have then been compared with reports from other countries and recommendations made to bring about more judicious antibiotic use in the Malaysian primary care setting.

## Methods

### Setting

Malaysia has a dual public-private health system with 871 public primary care clinics and 5198 private primary care clinics [11, 12]. Primary care functions as a partial gatekeeper to secondary care; people can bypass a referral from public or private practitioners and go directly to specialists and hospitals. The public healthcare system is heavily subsidised by the government; outpatient primary care entails a nominal payment of 1 Malaysian Ringgit ($0.25 USD) for consultations, investigations and medications. Private clinics are usually less crowded, have longer and more flexible opening hours, and are accessible through a variety of payment models (including out-of-packet payment, private insurance and coverage by third-party payers) [12]. A morbidity survey conducted in 2008 found that patients with chronic diseases were more likely to consult public clinics while private clinics cater mostly to preventive care and acute illnesses [13]. In 2008, public clinics accounted for 38 % of outpatient visits despite accounting for only 11 % of primary care clinics in Malaysia [14]. Malaysian healthcare reform is currently underway to improve the integration of public and private healthcare and to bring about more consistent clinical services and standard of care in both sectors [15].

### Data source and patient identification

The NMCS is a primary care survey conducted by the Clinical Research Centre Malaysia that collected information on clinical activities and prescriptions from primary care clinics in Malaysia. The study was conducted in 2014 and a detailed description of the NMCS methods is described elsewhere [16]. Briefly, NMCS is a national study of primary care activities whereby primary care clinics from public and private sectors in 14 states in Malaysia were sampled through stratified random sampling. The sampled clinics were required to provide records of patient encounters for a given date that was randomly assigned to each clinic. Doctors working on the survey day were asked to fill in a case report form to record information on patient demographics, reasons for encounter, diagnoses or problems managed, and interventions (medications, laboratory investigations, procedure/counselling, referral). Reasons for encounter, diagnoses and investigations were coded using the International Classification of Primary Care (ICPC-2) [17]. Medications were classified according to WHO Anatomical Therapeutic Chemical (ATC) codes [18]. Coding was performed by trained personnel and quality-control measures undertaken to ensure reliability of the data [16].

From NMCS records, we identified patients who were prescribed at least one systemic antibiotic. Systemic antibiotic were defined from the ATC code J01 (systemic antibacterials) and P01AB01 (oral metronidazole). Our primary outcome measure was antibiotic prescribing rate in public and private primary care clinics. We also determined the types of antibiotics prescribed and common diagnoses that resulted in prescription of antibiotics.

### Data analysis

Data were analysed and adjusted for the cluster study design using the survey function in STATA version 13 [19, 20]. Post-stratification weights were applied to the data to account for over or underrepresentation and non-response. Results were reported as the number of observations and percentages. The chi-square test was used to compare the antibiotic prescription rates in public and private clinics.

### Ethical approval

The NMCS received ethical approval from the Medical Research and Ethics Committee of the Ministry of Health Malaysia (NMRR-09-842-4718). A public notice was placed at each participating clinic to inform patients of the ongoing study and that data would be collected for research purposes only. Patients who do not want to be part of the study may opt out by informing the doctor and their data were not collected.

## Results

NMCS 2014 recorded 27,587 patient encounters from 545 primary care clinics. Of these, 5810 resulted in antibiotic prescription, corresponding to an overall prescribing rate of 21.1 % (Table 1). In 197 (3.4 %) of these encounters, more than one antibiotic was prescribed. The antibiotic prescribing rate was lower in public clinics (6.8 %) compared with private clinics (30.8 %). Private clinics accounted for approximately 60 % of all patient encounters, but around 87 % of all antibiotics prescribed in primary care.

**Table 1:** Summary of National Medical Care Survey (NMCS) 2014 dataset

	Public	Private	Overall
Total clinics	129	416	545
Patient encounters	11,172	16,415	27,587
Prescriptions^a^	9680	15,119	24,799
Total number of medications	27,762	45,417	73,178
Prescriptions containing antibiotics^b^	755	5055	5810
Total number of antibiotics (systemic)	767	5242	6009
Antibiotic prescribing rate^c^, %	6.76	30.79	21.06
Antibiotic per 100 patient encounters	7	32	22

^a^Each patient encounter in which medication was prescribed was counted as one prescription

^b^197 prescriptions (12 from public clinics, 185 private clinics) consisted of more than one antibiotic

^c^Percentage of encounters in which at least one antibiotic was prescribed

Table 2 shows the sociodemographic profiles of patients prescribed antibiotics. More female patients were given antibiotics in public clinics compared with male patients, while the reverse was true in private clinics. Antibiotics were mostly prescribed to patients aged 20–39 years. The distribution of the ten most common clinical diagnoses resulting in antibiotic prescription is presented in Table 3. Overall, acute upper respiratory tract infections (URTI) accounted for almost half of all antibiotics prescribed. Among patients with a diagnosis of URTI, 16.8 % were prescribed with antibiotics in public clinics whereas a higher prescribing rate was observed in private clinics (57.7 %). Private clinics prescribed more antibiotics for all ten diagnoses, in particular, URTI, fever (not otherwise classified), gastroenteritis and asthma.

**Table 2:** Characteristics of patient encounters prescribed with antibiotics

	Public	Private	Overall	
	*n* = 755	*n* = 5055	*n* = 5810	P-value^a^
Gender				
Male	309 (41.4 %)	2584 (51.8 %)	2893 (50.4 %)	*p* = 0.0005
Female	437 (58.6 %)	2407 (48.2 %)	2844 (49.6 %)	
Age group (year)				
< 1	26 (3.5 %)	74 (1.5 %)	100 (1.7 %)	*p* = 0.0001
1–9	159 (21.1 %)	774 (15.4 %)	933 (16.1 %)	
10–19	127 (16.9 %)	519 (10.3 %)	646 (11.2 %)	
20–39	231 (30.6 %)	2171 (43.2 %)	2402 (41.6 %)	
40–59	151 (20.0 %)	1145 (22.8 %)	1296 (22.4 %)	
≥ 60	59 (7.9 %)	342 (6.8 %)	401 (7.0 %)	
Education level				
No formal education	68 (12.4 %)	406 (10.8 %)	474 (11.0 %)	*p* = 0.0036
Primary	132 (24.3 %)	652 (17.3 %)	784 (18.2 %)	
Secondary	272 (49.9 %)	1726 (45.9 %)	1998 (46.4 %)	
Tertiary	73 (13.4 %)	975 (25.9 %)	1048 (24.4 %)	
Income level (MYR, per month)				
No income	324 (52.3 %)	1250 (32.5 %)	1574 (35.3 %)	*p* < 0.001
Less than 400	12 (2.0 %)	7 (0.2 %)	19 (0.4 %)	
400–999	63 (10.2 %)	259 (6.7 %)	322 (7.2 %)	
1000–1999	110 (17.8 %)	789 (20.5 %)	899 (20.1 %)	
2000–2999	56 (9.0 %)	697 (18.1 %)	753 (16.9 %)	
3000–3999	36 (5.8 %)	412 (10.7 %)	448 (10.0 %)	
4000–4999	10 (1.6 %)	199 (5.2 %)	209 (4.7 %)	
5000 and above	8 (1.4 %)	232 (6.0 %)	240 (5.4 %)	

Subtotal might not sum up to the total count because of missing values

^a^P-value from Chi-square test for comparison between public and private clinics, *p* < 0.05 considered as statistically significant

**Table 3:** Top ten diagnoses for prescription of antibiotics

		Public		Private		Overall	
Diagnosis	ICPC-2	n (%)	Antibiotic prescribing rate^a^	n (%)	Antibiotic prescribing rate^a^	n (%)	Antibiotic prescribing rate^a^
Upper respiratory infection, acute	R74	293 (38.7)	16.8	2564 (50.7)	57.7	2857 (49.2)	46.2
Acute tonsillitis	R76	97 (12.8)	83.3	254 (5.0)	92.7	350 (6.0)	89.9
Cystitis/urinary infection	U71	98 (13.0)	79.1	163 (3.2)	86.4	261 (4.5)	83.5
Gastroenteritis	D73	21 (2.8)	9.1	205 (4.1)	23.6	226 (3.9)	20.5
Fever	A03	12 (1.6)	3.6	202 (4.0)	32.3	213 (3.7)	22.4
Acute bronchitis/bronchiolitis	R78	4 (0.6)	45.3	96 (1.9)	57.7	100 (1.7)	57.0
Asthma	R96	11 (1.5)	3.1	63 (1.2)	14.8	74 (1.3)	9.5
Infectious disease, other^b^	A78	17 (2.3)	42.1	53 (1.0)	51.5	70 (1.2)	48.8
Boil/carbuncle	S10	18 (2.4)	79.4	49 (1.0)	93.9	68 (1.2)	89.4
Skin infection, Other^c^	S76	7 (0.9)	74.3	38 (0.8)	93.0	45 (0.8)	89.5

^a^Proportion of patient encounters for specific diagnosis that resulted in antibiotic prescription

^b^Diagnoses in this category included but not limited to abscess, sexually transmitted disease, bacterial infection

^c^Diagnosis in this category refers to cellulitis

The antibiotic groups prescribed in descending order of preference were: penicillins, cephalosporins, macrolides, quinolones and tetracyclines (Table 4). Prescriptions of more recently available broad-spectrum antibiotics such as quinolones, some macrolides (e.g., azithromycin) and lincosamides were more frequent in private clinics; these drugs were not held in the drug formulary of public primary care clinics. Most of these antibiotics were in oral dosage form with only 2.0 % prescribed as parenteral. For upper respiratory tract infections (URTI) and acute tonsillitis, the drug choices were understandably not so varied. However, the prescription of narrow-spectrum penicillin (phenoxymethyl penicillin) was rare, even for acute tonsillitis (Table 5).

**Table 4:** Types of antibiotics prescribed

	Public	Private	Overall
Antibiotic	n (%)	n (%)	n (%)
Penicillins	391 (51.0)	1451 (27.7)	1842 (30.7)
Amoxicillin	210 (27.4)	1037 (19.8)	1247 (20.8)
Cloxacillin	126 (16.4)	232 (4.4)	358 (6.0)
Bacampicillin	24 (3.1)	130 (2.5)	153 (2.6)
Ampicillin	24 (3.1)	46 (0.9)	70 (1.2)
Phenoxymethyl penicillin	5 (0.6)	2 (0.03)	7 (0.1)
Cephalosporins	76 (9.9)	1341 (25.6)	1417 (23.6)
Cephalexin	60 (7.9)	732 (14.0)	792 (13.2)
Cefuroxime	10 (1.4)	337 (6.4)	348 (5.8)
Cefadroxil	-	90 (1.7)	90 (1.5)
Cefaclor	-	73 (1.4)	73 (1.2)
Ceftriaxone	5 (0.7)	42 (0.8)	47 (0.8)
Cefoperazone, combinations	-	32 (0.6)	32 (0.5)
Macrolides	251 (32.7)	707 (13.5)	958 (16.0)
Erythromycin	251 (32.7)	357 (6.8)	607 (10.1)
Clarithromycin	-	164 (3.1)	164 (2.7)
Azithromycin	-	153 (2.9)	153 (2.5)
Roxithromycin	-	33 (0.6)	33 (0.6)
Penicillin, combinations	5 (0.7)	690 (13.2)	695 (11.6)
Amoxicillin and enzyme inhibitor	5 (0.7)	674 (12.9)	679 (11.3)
Quinolones	-	451 (8.6)	451 (7.5)
Ciprofloxacin	-	343 (6.6)	343 (5.7)
Ofloxacin	-	53 (1.0)	53 (0.9)
Tetracyclines	13 (1.7)	260 (5.0)	273 (4.6)
Doxycycline	13 (1.7)	246 (4.7)	260 (4.3)
Other antibiotics	20 (2.6)	161 (3.1)	181 (3.0)
Metronidazole	20 (2.6)	152 (2.9)	172 (2.9)
Sulfonamides & trimethoprim	11 (1.5)	113 (2.2)	124 (2.1)
Sulfamethoxazole and trimethoprim	11 (1.5)	111 (2.1)	122 (2.0)
Lincosamides	-	53 (1.0)	53 (0.9)
Lincomycin	-	34 (0.7)	34 (0.6)
Aminoglycosides	-	10 (0.2)	10 (0.2)
Carbapenem	-	5 (0.1)	5 (0.1)

**Table 5:** Type of antibiotics prescribed for upper respiratory tract infection and acute tonsillitis

	URTI			Acute tonsillitis		
	Public	Private	Overall	Public	Private	Overall
Antibiotic	n (%)	n (%)	n (%)	n (%)	n (%)	n (%)
Penicillins	129 (43.9)	794 (30.7)	923 (32.1)	47 (48.2)	53 (20.1)	99 (27.7)
Amoxicillin	102 (34.8)	658 (25.4)	760 (26.4)	41 (42.2)	43 (16.3)	83 (23.3)
Other penicillins	27 (9.1)	136 (5.3)	163 (5.7)	5 (5.6)	10 (3.8)	16 (4.4)
Cephalosporins	6 (2.0)	744 (28.8)	750 (26.0)	3 (2.6)	54 (20.6)	57 (15.8)
Cephalexin	6 (2.0)	454 (17.6)	460 (16.0)	2 (1.9)	24 (8.5)	26 (6.8)
Other cephalosporins	-	277 (10.7)	277 (9.6)	1 (0.7)	30 (11.6)	31 (8.7)
Macrolides	158 (54.1)	452 (17.5)	611 (21.2)	47 (49.2)	48 (18.2)	95 (26.6)
Erythromycin	158 (54.1)	253 (9.8)	412 (14.3)	47 (49.2)	19 (7.3)	67 (18.6)
Other macrolides	-	199 (7.7)	199 (6.9)	-	29 (10.5)	28 (7.7)
Penicillin, combinations	-	311 (12.0)	311 (10.8)	-	90 (34.4)	90 (25.1)
Quinolones	-	170 (6.6)	170 (5.9)	-	12 (4.4)	12 (3.2)
Other antibiotics	-	115 (4.4)	115 (4.0)	-	5 (1.9)	5 (1.4)
Total	293 (100)	2587 (100)	2880 (100)	97 (100)	262 (100)	359 (100)

*URTI* upper respiratory tract infection

## Discussion

In this study, data from a nationally representative sample of Malaysian primary care clinics revealed a high level of antibiotic prescribing in which at least one of five encounters resulted in prescription of an antibiotic. Antibiotic prescribing predominated in private clinics where they contributed 87 % of the total antibiotics prescribed in primary care. Notably, URTI, which is primarily viral in nature, accounted for half of all antibiotics prescribed. For most patients with respiratory tract infection, antibiotic use is unnecessary and these results suggest that antibiotics were not clinically indicated and are therefore overused in primary care clinics in Malaysia.

Excessive or suboptimal use of medicine in general, and antibiotics in particular, is a worldwide concern [21, 22]. A systematic review of medicine use in developing and transitional countries for the period 1990–2006 by the WHO reported that the overall antibiotic prescribing rate stabilised at 40–50 % while URTI-specific antibiotic prescribing rates has shown an increasing trend to 71 % in the period 2004–2006 [22, 23]. At first glance, the current Malaysian antibiotic prescribing rates (all encounters: 21.1 %, URTI encounters: 46.2 %) appear to be somewhat lower than the average rates from developing and transitional countries. However, the antibiotic prescribing rates in the review by WHO were generated using different methodologies from the present study and are therefore not entirely comparable.

The antibiotic prescribing rates in public and private clinics in our study were 6.8 and 30.8 %. A national morbidity and treatment survey of primary care clinics in 2008 revealed antibiotic prescribing rates in public and private clinics were 13.7 and 30.0 %, respectively (unpublished data, personal communication with Dr. Nordin Saleh, Ministry of Health, Kuala Lumpur, Malaysia). Thus, the antibiotic prescribing rate in public primary care clinics in Malaysia may be declining, while that of private clinics has remained relatively stable but at a high level. However, when compared with more developed countries, the antibiotic prescribing rates in Malaysia are still a cause for concern. For example, the URTI-specific antibiotic prescribing rate in Malaysia as seen in our study was 46.2 %, but the corresponding rates in the Netherlands and Hong Kong in 2010 were 17 and 5 %, respectively [24, 25].

Antibiotic prescribing rates in Malaysian primary care are much higher in private clinics compared with public clinics. Antibiotic over-prescribing (in private more than public clinics) has also been observed in surveys undertaken in Pakistan, India and several African countries [26–28]. In a systematic review of studies conducted in low- and middle-income countries by Basu et al., diagnostic accuracy and guideline adherence were more likely to be suboptimal in private primary care settings compared with the public sector [29]. The systematic review by Berendes et al., however, concluded that the quality of care from public and private primary care in low- and middle-income countries was generally of poor quality, although the private sector did perform better with regard to drug availability, responsiveness and client-centredness [30]. Our current survey highlights the problem of antibiotic overuse in Malaysian private primary care, both with respect to the quantity of excessive antibiotic prescribing as well as inappropriate antibiotic choice. Antibiotic prescribing in Malaysian private primary care was also noted to be excessive for several clinical conditions other than URTI, including acute bronchitis, acute gastroenteritis and asthma.

Higher antibiotic prescribing in private primary care clinics is partly owing to the higher proportion of acute self-limiting illnesses, such as viral fever and common colds. Furthermore, several reasons may explain the higher antibiotic prescribing rates in private clinics, among them that private general practitioners are more responsive to the patient’s expectation for antibiotics and the financial incentive of medication overuse, including that of antibiotics, as well as suboptimal knowledge about the aetiology of URTI and lack of awareness of the emergence of antibiotic resistance. Several local studies have documented misconceptions about the effectiveness of antibiotic therapy for URTI among the Malaysian general public and their healthcare providers, together with a lack of awareness of the harm of antibiotic resistance [9, 31–34].

Attempts to improve antibiotic prescribing practices in developing countries have been documented by several systematic reviews [35, 36]. These reviews have called for more multifaceted intervention studies over a longer time period to assess the sustainability of the interventions. A systematic review of antibiotic stewardship programmes in the outpatient setting included 50 studies, most conducted in developed countries, and showed that such programmes can improve antimicrobial prescribing without adversely affecting patient outcomes [37]. In Malaysia, antibiotic guidelines focusing on pharyngitis were introduced in 2003 [38] and were followed by national antibiotic guidelines in 2008 (updated in 2014) [39, 40]. It is possible that these clinical practice guidelines may have promoted more judicious antibiotic use among public primary care doctors. The current effort to improve antibiotic stewardship in Malaysia is still in the early stages [41, 42]. There remains a need to focus beyond the public health system to include the private healthcare setting as well. However, effective ways to bring about change in the latter remain unclear; systematic reviews have not identified any successful intervention programmes conducted in private primary care clinics [35–37]. It has been shown that more judicious antibiotic use in Malaysian primary care can occur if the right type of intervention is implemented, such as academic detailing by senior family physicians and feedback of prescribing data to primary care doctors [10, 43]. Public health education of the harm of excessive antibiotic use is currently being carried out in Malaysia and needs to be persistent [44, 45]. This is in keeping with reviews from high-income countries that suggest that a public health campaign can bring about more prudent antibiotic use [46]. Thus, by reaching out to the public at large, it is hoped that that patients will be less likely to insist on antibiotics whenever they consult primary care doctors for minor respiratory or febrile illness. In tandem with public health education, continuing medical education for practicing doctors needs to highlight the ongoing problem of antibiotic overuse and the emergency of antibiotic resistance. This has shown to be effective in Hong Kong where vocationally trained family physicians were less likely to prescribe unnecessary antibiotics [47].

### Strength and limitations of study

Our study has a number of important features. The large number of patient encounters obtained from clinics sampled across the country has allowed us to study clinical practice in primary care clinics. Moreover, our study used records from NMCS that collected data on patient visits in public and private clinics, allowing for comparison between the sectors. A limitation of the study is that the findings reflect practice observed in primary care settings; it does not include consultations within hospital outpatient or emergency departments. We also did not collect data from community pharmacies; hence, the supply of antibiotics to patients from community pharmacies was not considered. In Malaysia, antibiotic is not available over the counter and under the Malaysian Poisons Act the purchase of antibiotics requires prescription from physicians. However, dispensing separation has not yet taken place in Malaysia and very few prescriptions were filled at private pharmacies [48].

## Conclusions

This study has shown that antibiotics are frequently prescribed in primary care clinics and provides evidence of antibiotic prescribing for self-limiting conditions. Excessive and inappropriate antibiotic use in the Malaysian primary care setting, especially in private clinics, highlights the need for more concerted interventions targeting prescribers as well as general public. Improvement strategies should focus on reducing inappropriate prescribing. This should include simultaneous education of the public and healthcare providers via the mass media, professional societies and within the clinic setting. Improving prescribing habits in primary care is necessary to reduce the unnecessary use of antibiotics and limit subsequent antibiotic resistance.

### Ethics and consent to participate

This study was reviewed and approved by the Medical Research and Ethics Committee of the Ministry of Health Malaysia. A public notice was placed at each participating clinics to inform patients of the ongoing study and patients who do not wish to participate may opt out from the study. Aggregate data was used, with no identifying personal details published.

### Consent to publish

Not applicable.

### Availability of data and materials

All the data supporting the findings are available within the manuscript, additional data available upon request.

## Abbreviations

ATC: Anatomical Therapeutic Chemical
ICPC: International Classification of Primary Care
NMCS: National Medical Care Survey
URTI: upper respiratory tract infection
WHO: World Health Organization
